# Rapid Creation of Interspecific Hybrid Progeny to Broaden Genetic Distance through Double Haploid (DH) Inducer in *Brassica napus*

**DOI:** 10.3390/plants11050695

**Published:** 2022-03-04

**Authors:** Ying Zhou, Meicui Yang, Shihui Zhao, Haoran Shi, Yun Li, Wanzhuo Gong, Jin Yang, Jisheng Wang, Qiong Zou, Lanrong Tao, Zeming Kang, Rong Tang, Shixing Guo, Shaohong Fu

**Affiliations:** 1 Chengdu Academy of Agriculture and Forestry Sciences, Chengdu Research Branch, National Rapeseed Genetic Improvement Center, Chengdu 611130, China; 1357226131@163.com (Y.Z.); 2533684145@163.com (M.Y.); yoril@foxmail.com (S.Z.); 18697480039@163.com (H.S.); 83306710@163.com (Y.L.); 501116@163.com (W.G.); yjjing@163.com (J.Y.); 136681806522@163.com (J.W.); 382670439@163.com (Q.Z.); 382668280@163.com (L.T.); 13684001789@163.com (Z.K.); 1127427933@163.com (R.T.); 2 College of Agriculture, Sichuan Agricultural University, Chengdu 611130, China

**Keywords:** double haploid induction line (DH inducer), interspecific hybridization, genetic distance, fluorescence in situ hybridization (FISH)

## Abstract

Interspecific hybridization of rapeseed is an important way to innovate breeding resources. This research used *Brassica napus* and *Brassica rapa* for artificial synthesis interspecific hybridization of F_1_. The F_1_ self-fruiting rate was particularly low. By comparing the fertilization rate and seed setting rate of nine crosses and selfing combinations of interspecific hybrid progeny F_1_ and control *B. napus*, the results proved that the genetic stability of egg cells was greater than that of sperm cells, so the F_1_ could get seed by artificial pollination with other normal pollen. Based on these results, interspecific maternal inbred offspring (induced F_1_) from egg cells was obtained by emasculation and pollination with the pollen of DH inducer Y3380. It was found through morphological analysis, flow cytometry identification, and meiotic observation of induced F_1_, the plants had most normal fertile tetraploid and the meiosis was normal. The FISH results showed that the induced F_1_ were *B. napus* (2n = 4x = 38, AACC), 20 A and 19 C chromosomes. The results of SNP chip detection and genetic cluster analysis found that the genetic variation between interspecies could be preserved or broadened in the induced F_1_. The use of DH inducer created special breeding resources for interspecific hybridization and distant hybridization of rapeseed while shortening time, improving efficiency, and providing a new insight into innovate breeding resources.

## 1. Introduction

Distant hybridization between *Brassica* species or between closely related genera is one of the most important means to innovate the resources of *B. napus*. It can help to improve the traits of *B. napus*, such as the hybrid offspring was rich in genetic diversity and higher yield [1,2], broaden the genetic basis of disease resistance in *B. napus* [3], create a new yellow seed germplasm of *B. napus* [4], significantly increase the protein and oil content and the content of essential amino acids [5], breed a new germplasm with long and dense siliques [6], and increase nitrogen nutrient utilization and tolerance to low nitrogen stress of *B.* *napus,* etc. [7]. However, the lack of seed setting or low seed setting rate caused by hybrid sterility [8] and chromosomal mismatch in the offspring of interspecific hybrids of rapeseed is a problem that needs to be solved urgently. To solve this problem, the offspring of interspecific hybridization are usually backcrossed continuously with their parents to increase the seed setting rate [9], but continuous backcrossing can cause the genetic distance to be closer and closer to the parents [10], and the selection cycle of this method could be lengthened. At present, microspore culture has been widely used to develop homozygous lines [11,12,13,14], but microspore culture is vulnerable to the influence of genotype [15] and culture conditions [16,17]. Microspore culture technology is difficult to use in interspecific hybrid breeding because the microspore fertility of interspecific hybridization F_1_ is low and there are other methods that cannot only shorten the breeding period, but perhaps can maintain the genetic distance of hybrid offspring.

In recent years, Fu [18,19] synthesized and bred a synthetic octoploid rapeseed, e.g., Y3560 and Y3380 (2n = 8x ≈ 76, AAAACCCC) in the breeding process of rapeseed. When this octoploid was used as a pollen donor to pollinate different genetically stable *Brassica* crops (*Brassica* *oleracea* and *Brassica napus*) [20], a large number of homozygous individuals similar to the female plant appeared in the offspring. Later, it was confirmed by field performance, flow cytometry, and SNP chip technology that the phenotype, ploidy, and genotype of these offspring were consistent with those of the female parent. This showed that maternal induction occurred in this pollination process. To describe the characteristics of the induction material more accurately, this method was named the double haploid (DH) inducer, e.g., Y3560 and Y3380 in *B. napus*, referred to as DH induction line (DH inducer). Through the observation of the induction phenomenon, the phenotype of the offspring, and the detection of SNP chip, it was speculated that the induction process was due to the zygotes produced by the inducible sperm cells and the maternal parent eggs, the paternal chromosome being eliminated during the mitosis of the zygote, and then the maternal chromosome doubling and forming DH offspring [21,22]. Can use of DH inducer pollen instead of interspecific F_1_ continuous backcrossing with the parent to double the chromosomes of interspecific F_1_ egg cells form induced offspring with genetic diversity? Therefore, this study used the hybridization of *B. napus* (AACC, 2n = 4x= 38) and *B. rapa* (AA, 2n = 2x = 20) to obtain F_1_. By comparing the genetic stability of the pollen mother cell and the egg cell of F_1_, it was found that the genetic stability of the egg cell of the interspecific hybridization was greater than the sperm cell. The results laid a theoretical foundation for the induction of interspecific hybrid F_1_ by DH inducer to obtain normal offspring. The progeny of the interspecific hybrid F_1_ induced using the DH inducer was called the induced F_1_. The chromosome composition was analyzed by chromosome fluorescence in situ hybridization, and the homozygous site rate of induced F_1_ and the genetic distance from the parent were analyzed by SNP to determine whether induced F_1_ was stable new rapeseed, and whether its genetic variation had broadened. The research aims to solve the shortcomings of the long cycle of creating new germplasm in rapeseed, and to provide new insights and methods for rapeseed resource innovation.

## 2. Results

### 2.1. Phenotype, Fertility, and Flow Cytometric Identification of Interspecific Hybrid F_1_ and Induced F_1_ Plants

**Figure 1:**
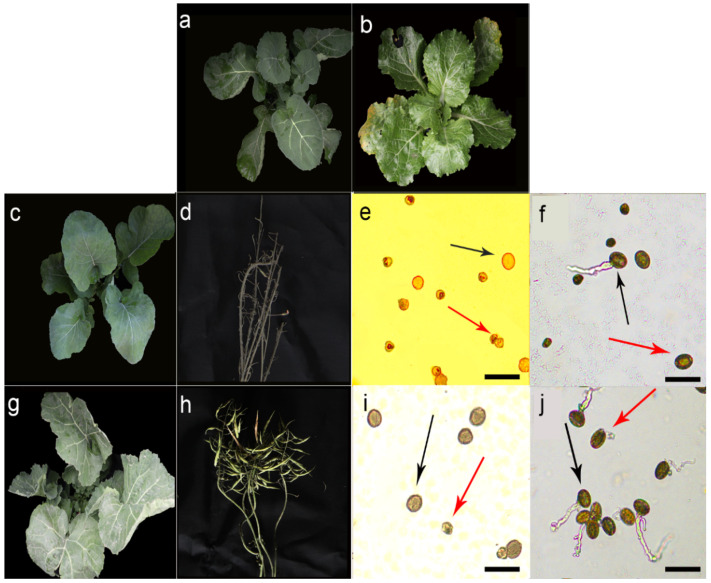
Phenotype observation diagram of some parent, F_1_, and induced F_1_ plants: (**a**) ZS11 seedling stage morphological characteristics; (**b**) HZ32 seedling stage morphological characteristics; (**c**) T19-5 seedling stage morphological characteristics; (**d**) T19-5 mature fruit pods; (**e**) T19-5 pollen grain morphology observation, black arrows indicate fertile pollen grains, red arrows indicate sterile pollen grains; (**f**) T19-5 pollen germinates after 4 h in vitro observation, the black arrow points to the germinated pollen grains, and the red arrow points to the ungerminated pollen grains; (**g**) W59 seedling morphological characteristics; (**h**) W59 mature fruit pods; (**i**) W59-8 pollen grain morphology observation, black arrows indicate fertile pollen grains, red arrows indicate sterile pollen grains; (**j**) W59 observation of pollen tube germination 4 h after the pollen was in vitro, the black arrow points to the germinated pollen grains, and the red arrow points to the ungerminated pollen grains. Ruler = 20 μm.

P3-2 and ZS11 (*B. napus*, AACC, 2n = 4x = 38) were used as female parents, and Y6 and HZ32 (*B.* *rapa*, AA, 2n = 2x = 20) as male parents, respectively, and interspecific hybridizations were carried out (Table S1). Morphological observation of F_1_: The plants of T19-4 and T19-5 had normal vegetative growth (Figure 1c), their morphological characteristics were similar to those of the maternal parent *B. napus* (Figure 1a,b), and the self-bred siliques had no obvious development and grain filling (Figure 1d). The average self-bred seed setting rates of T19-4 and T19-5 were 4.00% and 5.19%, respectively (Table 1). Therefore, the pollen grains of parents, T19-4 and T19-5, were observed by staining. The normal pollen morphology proportions of parents *B. napus* P3-2, ZS11 and *B. rapa* Y6 and HZ32 were 94.19%, 98.51%, 97.20%, and 98.70%, respectively (Table S2). After the pollen of P3-2, ZS11, Y6, and HZ32 was cultured in-vitro for 4 h, the pollen tube germination rate was calculated. The average pollen tube germination rate was 21.08%, 34.12%, 34.07%, and 32.97% (Table S2), respectively. The normal pollen morphology proportions of T19-4 and T19-5 were 30.24% and 11.14%, respectively (Figure 1e, Table S2). The pollen tube germination rates of T19-4 and T19-5 pollen after 4 h in vitro were further calculated. The average pollen tube germination rates of T19-4 and T19-5 were 5.85% and 1.29%, respectively (Figure 1f, Table S2), indicating that the pollen fertility and pollen germination ability might be the main reasons for the low selfing rate. To achieve the seed setting of interspecific hybrids F_1_, we pollinated hybrid F_1_ generations T19-4 and T19-5 with double haploid induction line Y3380 and observed the morphology of induced F_1_ generations W58 and W59 (Figure S1). The morphological characteristics of the plants were partial to the female parent *B. napus* (Figure 1a,b,g), they were rich in morphological features (Figure S1): The leaf shape was diverse, the leaf color was different, and it was an early maturing single plant, and so on. The siliques were enlarged and elongated, and the length of the siliques was longer than the siliques of T19-4 and T19-5 (Figure 1d,h). The statistical results of the average self-bred seed setting rate of W58 and W59 were 29.33% and 39.13% (Table 1). The proportions of normal pollen morphology of W58 and W59 were 92.62% and 95.89%, respectively (Figure 1i, Table S2). The pollen tube germination rate of W58 and W59 after 4 h in vitro was counted, and the results were 26.84% and 23.59%, respectively (Figure 1j), which were significantly higher than the F1 generation of interspecific crosses, and the selfing results were normal.

**Figure 2:**
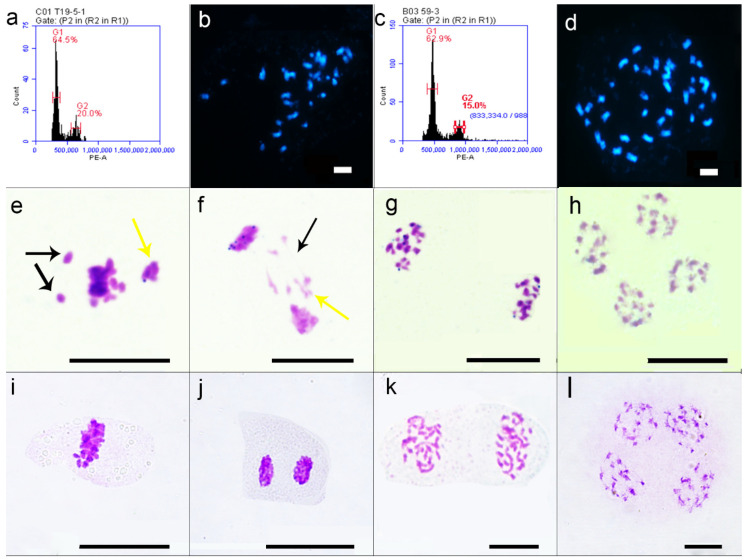
Part of F_1_ and induced F_1_ flow cytometry and observation of ordinary cells: (**a**) T19-5 flow cytometry diagram; (**b**) T19-5 root tip mitosis observation diagram; (**c**) W59 flow cytometry diagram; (**d**) W59 root tip mitosis observation diagram; (**e**) T19-5 meiosis prophase I, the black arrow points to the monovalent body, the yellow arrow points to the multivalent body; (**f**) T19-5 meiosis anaphase I, the black arrow points to the chromosome bridge, and the yellow arrow pointing to the chromosome bridge refers to lagging chromosomes; (**g**) T19-5 post meiotic stage I; (**h**) T19-5 tetrad period; (**i**) W59 meiotic prophase I; (**j**,**k**) W59 post meiotic stage I; (**l**) W59 tetrad period. Ruler = 10 μm.

At the same time, the parent, F_1_, and induced F_1_ seedling stage were identified by flow cytometry, with tetraploid *B. napus* ZS11 used as a control. The results of flow cytometry analysis can be seen in Table 2 and Table S3. The G1 peak of tetraploid *B. napus* ZS11 was 495.89–510.69 D thousand lines, and the G1 peak of P3-2 was 482.62–498.71 D thousand lines, verifying that it was a tetraploid; the G1 peak of Y6 was 261.16–313.41 D thousand lines, and the G1 of HZ32 The peak value was 243.76–303.69 D thousand lines, verifying that Y6 and HZ32 were diploid. The G1 peak of 27 plants of T19-4 was 312.12–482.46 D thousand lines, of which one plant was judged to be tetraploid, and the remaining 26 plants were triploid. The G1 peak of 23 plants of T19-5 was approximately 307.29–493.68 D thousand lines, as shown in the figure; the G1 peak of T19-5-1 was 322.84 D thousand lines (Figure 2a), of which one plant was judged to be tetraploid, and the remaining 22 plants were triploid. The two tetraploid F_1_ of T19-4 and T19-5 plants may be pseudo-hybrid. Flow analysis of all induced F_1_ was performed as follows: The G1 peak of 18 plants of W58 lines was 421.45–661.17 D thousand lines, as shown in the figure; the G1 peak of W59-3 was 478.27 D thousand lines (Figure 2c), of which 15 were tetraploid plants; the G1 peak of six plants of W59 lines was 478.27–1037.64 D thousand lines, of which three were tetraploid plants. Among W58 and W59, a total of five hexaploids and one octoploid were found. The results show that the induced F_1_ produced by the interspecific hybrid F_1_ and the DH inducer Y3380 could produce tetraploid rapeseed with normal pollen fertility, pollen vigor, and seed self-bred setting rate.

### 2.2. Cytological Observation of Interspecies Hybrid F_1_ and Induced F_1_

After phenotypic and flow cytometric analysis of the interspecific hybrid F_1_ and the induced F_1_, the root tip mitosis was further observed, and it was found that after removing the pseudohybrid plants, both T19-4 and T19-5 had 29 chromosomes (Figure 2b). The mitotic results of W58 and W59 plants were verified to have 38 chromosomes (Figure 2d), which was consistent with the tetraploid detected by flow cytometry. Observation of meiosis found that there were serious abnormalities in the meiosis process of T19-4 and T19-5: More chromosomal abnormalities were observed, such as chromosome bridges, chromosome backwardness, and unequal division; monovalent, bivalent, and multivalent bridges could be seen in metaphase I of meiosis (Figure 2e). The number of bivalents varied between two–seven, however the cell frequency of six and seven bivalents was higher, and their average pairing configuration was 4I + 5.29II + 2.71III + 1.57IV, indicating that not only do homologous associations occur during meiosis, but also heterologous associations of non-homologous chromosomes between the A and the C genome. At the later stage of meiosis I, the number of lagging chromosomes ranged from one to seven (Figure 2f). In the later stage of meiosis I, the cells separated abnormally, with more chromosomes at one pole and less chromosomes at the other pole (Figure 2g). The number of chromosomes separated to the two poles of cells was 12/13, 12/15, 12/17, 13/14, and 12/17, except for the number of chromosomes that divided into two cells on 12/17 and 14/15, which were 29. The other division methods were less than 29, indicating that chromosomes were lost during the process of meiosis I. In the process of meiosis II, there were still abnormal phenomena such as laggard chromosomes and unequal division. The total number of chromosomes divided into tetrad period cells was less than 58 (Figure 2h). This further shows that the disorder of meiosis in triploid T19-4 and T19-5 pollen mother cells (PMCs) could not produce normal gametes, but more aneuploid gametes, which leads to low pollen fertility, low pollen vigor, and extremely low selfing seed setting rate or inability to complete selfing.

The pollen mother cells (PMCs) of induced F_1_ tetraploid W58 and W59 were observed through pollination of interspecific hybrid F_1_ emasculation by DH inducer Y3380: The meiosis of W58 and W59 was normal, and there was no chromosomal lag in metaphase I (Figure 2i). The chromosomes were paired normally, and the number of bivalents was 19, with no monovalent and polyvalent bodies (Figure 2i); the cells in late meiosis I were normally separated, and no imbalance of chromosome bridge and chromosome division was observed. The number of chromosomes separated to the cell poles was 19/19 (Figure 2k). During meiosis II, there was no chromosome bridge and unequal chromosome division, and the division was normal in the tetrad stage (Figure 2l). The meiosis of pollen mother cells (PMCs) of W58 and W59 could produce normal gametes so that the pollen fertility was high, the pollen vigor was strong, and the seed setting rate was also normal. This indirectly proved that F_1_ female gametophytes (egg cells) of interspecific hybridization could normally produce tetraploid progeny induced by induction lines, also indicating that the heredity of female gametophytes was more stable than that of male gametophytes.

### 2.3. Observation of Germination, Fertilization Rate, and Seed Setting Rate in Interspecific Hybrid F1 Pollen Tube

**Figure 3:**
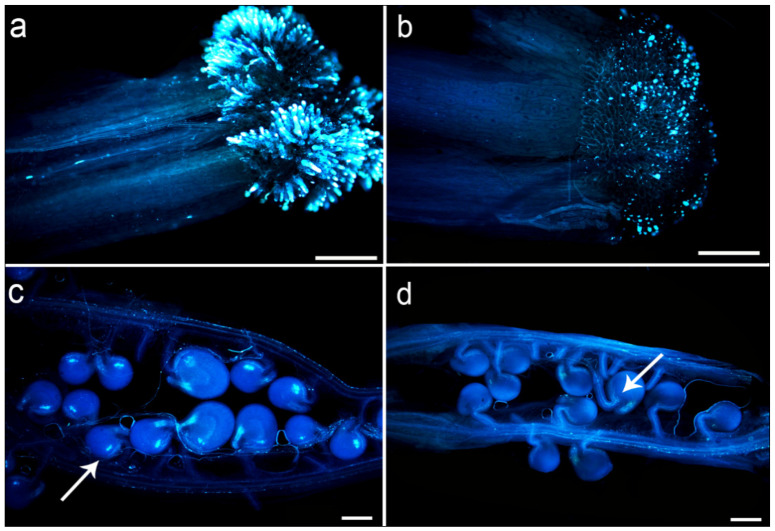
The adhesion of the stigma of each hybrid combination to pollen after 24 h of pollination and the observation of pollen tube extension in the silique after 48 h of pollination: (**a**) Y3380 pollen was pollinated on T19-5 stigma; (**b**) T19-5 pollen was pollinated on T19-5 stigma. (**c**) The extension of the pollen tube in the silique after the Y3380 pollen was pollinated on the T19-5 stigma for 48 h, the arrow indicates that the pollen tube entered the bead hole and complete fertilization. (**d**) The extension of pollen tube in the silique after pollination of T19-5 pollen on the stigma of T19-5 for 48 h. The arrow indicates that the pollen tube entangled and piled up near the bead hole and could not enter the bead hole to complete fertilization. Ruler = 1 mm.

As the egg cells were not easy to separate and the number of egg cells was sufficient, they were difficult to use for cytological research. It was necessary to determine how to compare the genetic stability of sperm and egg cells with interspecific hybrids of rapeseed. In this study, we observed the pollen adhesion of nine cross combinations of T19-4 and T19-5 after pollination for 24 h. By comparing the fluorescence intensity of the stigma with the amount of fluorescence, the results showed that the fluorescence intensity of the pollen of the inducer lines Y3380 and ZS11 at the stigma of T19-4, T19-5, and ZS11 was stronger (Figure 3a), and pollen could adhere well to the stigma. However, the pollen of T19-4 and T19-5 had only a small amount of fluorescence on their respective styles (Figure 3b). The style of control of ZS11 showed that the germination rate of triploid pollen was low, and it was difficult for a large number of pollen tubes to enter the ovary. After 48 h of pollination, the pollen tube extending into the ovary was further observed: Except for T19-4 and T19-5 selfing (Figure 3d), a large number of pollen tubes in other combinations entered the ovule. In the center of the ovule, there was a significant fluorescence (Figure 3c), which indicated that the ovule had been fertilized. Pollen tubes of T19-4 and T19-5 selfing entangled and piled around the ovule and could not enter the ovule to complete fertilization (Figure 3d), eventually leading to ovule abortion and failure to form seeds. By comparing the average fertilization rate and the average seed setting rate of the nine combinations (Table 1), it was found that the self-fertilization rates of T19-4 and T19-5 were 6.25% and 11.90%, respectively. After T19-4 and T19-5 were emasculated and pollinated with ZS11 pollen, the average fertilization rates were 60.34% and 69.23%, respectively. On the contrary, when ZS11 was used as a female parent and pollinated with T19-4 and T19-5, the fertilization rate dropped to 23.26% and 26.47%, which further showed that the main reason why interspecific hybrid F_1_ could not self-bred was that the germination rate of triploid pollen was low and could not germinate normally in the stigma, therefore the pollen tube could not enter the ovule to complete fertilization. However, using the DH inducer Y3380 pollen to pollinate T19-4 and T19-5, the fertilization rate and the seed setting rate were much higher than those with self-pollen. This also confirmed why T19-4 (T19-5) × Y3380 could obtain a large number of induced progenies.

### 2.4. Interspecific Hybridization F_1_ and Induced F_1_ Fluorescence In Situ Hybridization

**Figure 4:**
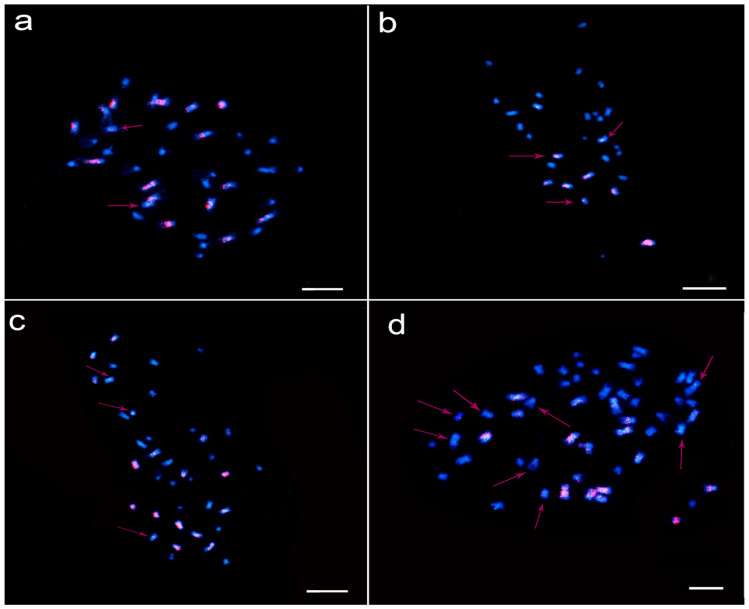
FISH diagram of partial F_1_ and induced F_1_. (**a**) ZS11 FISH pictures; (**b**) T19-4 FISH pictures; (**c**) W58-9 FISH pictures; (**d**) W59-2 FISH pictures. The specific *Brassica* C genome sequence (DNA BNIH123L05) is indicated by the red signal, and A genome has no red signal. Some C chromosomes with weak red fluorescence are indicated by red arrows. Ruler = 20 μm.

Through DH inducer and interspecies crossing F_1_ emasculation and pollination, induced F_1_ offspring were obtained. From the chromosome number and flow cytometric identification, it was proven that part of induced F_1_ was tetraploid, but it was unclear whether the constitution of chromosomes A and C was consistent with that of normal *B. napus*. In order to obtain the chromosomal constitution of interspecific hybridization F_1_ and tetraploid-induced F_1_, fluorescence in situ hybridization was performed on T19-4, T19-5, W58, and W59, and ZS11 was used as a control. The result showed that ZS11 had 38 chromosomes, 18 C genome chromosomes (chromosomes had red signals), and 20 A genome chromosomes (no red signal) (Figure 4a). There were 29 chromosomes in F_1_ (T19-4) of interspecific hybridization, nine chromosomes of C genome (chromosomes had red signals), and 20 chromosomes of A genome (no red signal) (Figure 4b). The configuration of F_1_ chromosome of interspecific hybridization was AAC (2n = 3x = 29). The result of induced F_1_ was the same as that of ZS11, with a total of 38 chromosomes: 18 C genome chromosomes (chromosomes had red signals), and 20 A genome chromosomes (no red signal) (Figure 4c). Therefore, the induced F_1_ offspring of the interspecific hybrid triploid F_1_ (2n = 3x = 29, AAC) induced by the DH inducer Y3380 was a tetraploid *B. napus* (2n = 4x = 38, AACC). This meant that the matched structures of chromosomes 20 A and 18 C were relatively stable during induction. In addition, we selected some hexaploid plants from crossing by interspecific hybridization F_1_ and octoploid induction line Y3380 to observe by fluorescence in situ hybridization: Hexaploid W58 and W59 had about 54 chromosomes with 27 C genome chromosomes (chromosomes had red signals), and 27 A genome chromosomes (no red signal) (Figure 4d).

### 2.5. Homozygous Rate and Genetic Distance Cluster Analysis of Induced F1 Generation SNP Loci 

**Figure 5:**
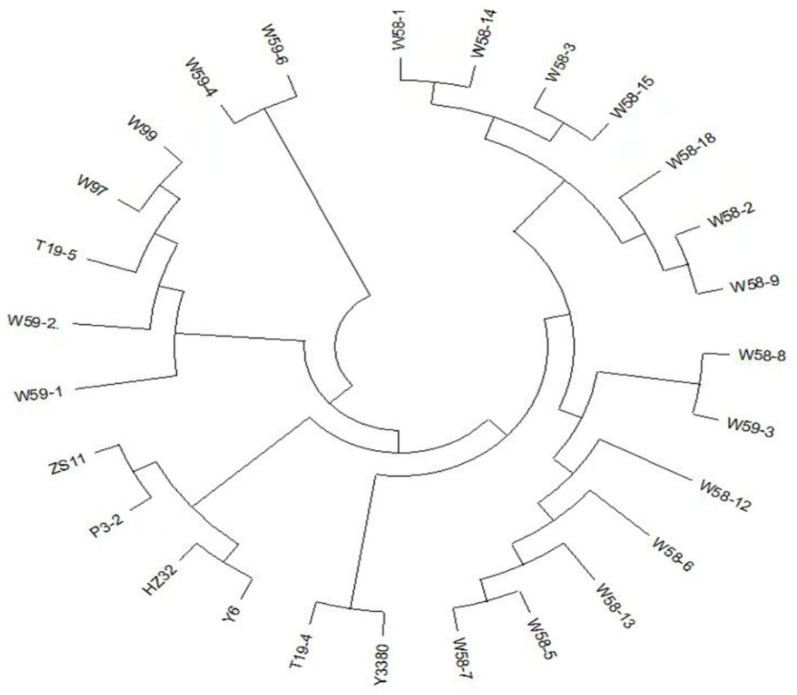
Genetic cluster analysis of parents, F_1_, F_2_, BC_1_F_1_, induced F_1_, the double haploid (DH) inducer. Parents: P3-2, ZS11, Y6, HZ32, F_1_: T19-4, T19-5; F_2_: W97, BC_1_F_1_: W99; induced F_1_: W58, W59; the double haploid (DH) inducer: Y3380. Among W58 and W59, except W58-9, W58-12, and W59-2 were hexaploid plants, W59-1 was octoploid plants, and the rest were tetraploid plants.

In order to determine the genetic stability and genotypic homozygous rate of the induced F_1_ generation, the surviving 13 strains of W58 line and five strains of W59 line were analyzed by 50 k SNP chip. In total, 42,090 effective sites were detected, classified, and counted as AA, BB, AB, respectively. AB represented the heterozygous site; AA and BB were the homozygous sites; and the percentage of homozygous sites was counted. The homozygous rate per plant of W58 line was 74.24%–81.63% (Figure S2, Table S4), the homozygous rate of all plants in W58 line was greater than 70%; and the homozygous rate of the individual plant of W59 was 58.93%–78.62% (Figure S2, Table S4). The F_1_ generation of interspecific hybridization induced by the DH inducer Y3380 could quickly select more homozygous sites and provided a higher homozygous rate (greater than 70%) for a stable single plant. In general, the homozygous rate of hybrid offspring was about 60%. For the parents, by comparing F_1_, F_2_, induced F_1_, BC_1_F_1_, differences in SNP loci between the two materials, an *n* × *n* matrix could be obtained. The smaller the difference, the smaller the value of the matrix elements, and the closer the distance between the two samples. Thus, all samples were effectively clustered together, and a cluster diagram of the genetic distance of the samples was drawn (Figure 5) (due to the death of W94 and W96 plants, there was no T19-4 selfing progeny and backcross to the offspring’s data). The genetic distance between T19-4, T19-5, W58, and W59 was further from the parent and fell in a cluster class different from the parent. The genetic distance between T19-5 induced progeny W59 and parents was further than that of T19-5, T19-5 self-crossing progeny W97, T19-5 backcrossing progeny W99, and parents. All plants in W58 fell on a cluster class different from T19-4, namely all plants had rich genetic variation and were developing in a different evolutionary direction from T19-4. The three plants in W59 line (W59-3, W59-4, W59-6) were not in the same cluster class as T19-5, which indicated that the three individual plants in the W59 line were also rich in genetic variation. Therefore, the induction of interspecific hybrid offspring of rapeseed by the DH inducer could not only stabilize the induced offspring, but also preserve or broaden the genetic distance of some individual plants. The use of DH induction lines not only achieved the acquisition of self-progeny of F_1_ megaspores of interspecific hybrids, but also maintained the purpose of expanding genetic distances of interspecific hybrids from the analysis of SNP and genetic distance.

## 3. Discussion

### 3.1. The Reasons for the Low Seed Setting Rate of Interspecific Hybridization between B. napus and B. rapa and the Genetic Stability of Egg Cells Provides the Possibility for DH to Induce Egg Cells

The hybrid sterility of interspecific hybridization F_1_ was obvious [8], and there were chromosomal mismatches. Research on the cytological level was limited to the genetic analysis of pollen mother cells (PMCs) [23,24,25]. Through the observation of meiosis, pollen fertility investigation, pollen tube germination ability, and fertilization rate of F_1_ pollen mother cells (PMCs), it was found that the hybrid offspring of *B. napus* (AACC, 2n = 4x = 38) and *B. rapa* (AA, 2n = 2x = 20) T19-4 and T19-5 have abnormal meiosis. This resulted in low pollen fertility and low pollen vigor [26]. In addition, the low pollen fertility and low pollen germination rate resulted in only a small amount of pollen attached to the style for germination (Figure 3b); a part of the pollen tube that entered the embryo sac entangles and accumulates near the bead hole and could not enter the bead hole to complete fertilization (Figure 3d), resulting in low self-fertilization rate and low self-seed setting rate (Table 1). It was difficult to directly use the pollen of the hybrid offspring of *B. napus* and *B. rapa* for self-pollination to obtain a large number of stable genetic offspring.

Although the genetic analysis of pollen mother cells was extensive [23,24,25], the genetic analysis of egg cells had not been reported. In an attempt to obtain induced offspring by inducing interspecies hybridization F_1_ through DH induction lines, the key question was whether the interspecies hybridization F_1_ megaspore mother cells could produce normal megaspore gametes. In order to determine whether the megaspore mother cell had abnormal meiosis and whether it could be fertilized normally, the normal tetraploid pollen was crossed with the interspecific cross F_1_ (triploid), the ovule was stained with aniline blue to determine whether the ovule was fertilized, and the seed setting rate was analyzed (Table 1). The average seed setting rate of ZS11 × T19-4, ZS11 × T19-5 was 11.48%, 7.62%, the average fertilization rate of selfing with ZS11 was 90.56%, and the average seed setting rate of ZS11 selfing was 50.25%. It could be seen that the average fertilization rate and the average seed setting rate of ZS11 × T19-4 and ZS11 × T19-5 were significantly reduced, which further indicated that abnormal F_1_ pollen meiosis led to aggravation of pollen aneuploid gametes, pollen abortion, and low pollen vigor. In addition, due to the low pollen fertility of F_1_ and the low rate of pollen tube germination, the F_1_ pollen also had less fluorescence on the ZS11 stigma, which made the average fertilization rate and average seed setting rate of ZS11 × T19-4 and ZS11 × T19-5 significantly lower than the average fertilization rate and average seed setting rate of ZS11 selfing.

The average fertilization rate and average seed setting rate of T19-4 × ZS11 and T19-5 × ZS11 were higher than the average fertilization rate and average seed setting rate of T19-4 and T19-5 selfing. When using normal pollen ZS11 to pollinate T19-4 and T19-5, the average fertilization rate and the average seed setting rate were significantly improved, indicating that with T19-4 and T19-5 egg cell inheritance the stability was far greater than the genetic stability of T19-4 and T19-5 sperm cells. Using T19-4 and T19-5 as the female parent, other plants with normal pollen such as the male parent could theoretically bear fruit normally. This was also an important reason for interspecific hybrid F_1_, using hybrid F_1_ as the female parent and backcrossing the parent to obtain offspring [9]. At the same time, this result laid a foundation for the DH inducible line to induce the interspecific hybrid F_1_ to obtain egg cell self-bred progeny.

Through pollination of interspecific hybrid F_1_ by DH inducer Y3380, the average fertilization rate of T19-4 × Y3380 and T19-5 × Y3380 was 83.93% and 73.68%, and the average seed setting rate of T19-4 × Y3380 and T19-5 × Y3380 was 26.46% and 20.44%. This was much higher than the average fertilization rate of T19-4 and T19-5 selfing of 6.25% and 11.90%, and the average seed setting rate of T19-4 and T19-5 of 4.00% and 5.19%. Therefore, this result proved that the meiosis of interspecific hybrid F_1_ egg cells was relatively normal, and its genetic stability was stronger than that of sperm cells. It has become a reality to use DH inducers to induce interspecies hybrid F_1_ to obtain egg cell self-progeny.

### 3.2. Ploidy Changes and Cytological Genetic Stability of Induced F1 Progeny by Interspecific Hybridization

The induced F_1_ by interspecies hybridization was obtained by induction selfing through F_1_ egg cells [21,22], and there might be changes in the number of chromosomes and ploidy changes. Through the analysis of the ploidy of T19-4 and T19-5, T19-4 and T19-5 were mostly triploid (excluding two pseudohybrid). Through observing the meiosis of its pollen mother cell, it was found that meiosis was abnormally disordered, and this is the reason for low pollen fertility and low pollen germination rate. If the meiosis of the megaspore mother cell was as chaotic as the meiosis of the pollen mother cell, there would be obvious ploidy and chromosome number change for the offspring that induced selfing of the egg cell. Ploidy analysis of the lines produced by W58 and W59 found that there were 5 of 24 hexaploid plants and 1 of 24 octoploid plants, and the rest were judged to be tetraploid by flow cytometry. The chromosome number of the tetraploid plant was 38 (Figure 2d). Through cytological identification and FISH observation, it was found that hexaploid plants have about 54 chromosomes (2n = 6x ≈ 54, AAACCC) (Figure 4d), analyzing the source of hexaploid plants. Since the octoploid DH inducer was prone to producing aneuploid gametes [22,27], it was basically determined that it was the interspecific hybrid F_1_ and the octaploid DH inducer hybridized which lost three A chromosomes. It was inferred that the octoploid plants in W59 were also interspecific crosses between F_1_ and octoploid DH induction lines. The tetraploid produced after induction was observed by meiosis and there was no abnormality, indicating that the tetraploid produced after induction was cytologically and genetically stable. In order to determine the configuration of T19-4, T19-5, W58, and W59 chromosomes, FISH analysis was performed: The results confirmed that the configuration of T19-4 and T19-5 chromosomes was triploid rape (2n = 3x = 29, AAC) (Figure 4b). Tetraploid plants W58 and W59 were normal tetraploid *B. napus* (2n = 4x = 38, AACC) (Figure 4c). This result further showed that the egg cells of the hybrid offspring of *B. napus* and *B. rapa* were prone to form normal gametes, with 10 A chromosomes and nine C chromosomes after meiosis. This also showed that the synthesis of *B. napus* 7500 years ago was not accidental [28].

Therefore, through the observation of the ploidy change and FISH of the progeny induced by interspecific hybridization, we found that after the interspecific hybrid F_1_ was induced by the inducible line Y3380, it was more likely to form normal tetraploid *B. napus* (2n = 4x = 38, AACC). Again, this proved that the meiosis of the egg cells of interspecific hybrid F_1_ were relatively normal, and it was easy to form normal gametes.

### 3.3. The F_1_ Progeny Produced by the Interspecific Hybridization Retains the Rich Genetic Variation of B. napus and B. napus

Interspecific hybridization could broaden the germplasm resources of rapeseed and is an important way to utilize excellent specific genes [9,29,30]. However, due to the mismatch of the number of chromosomes, the chaotic meiosis of pollen mother cells (PMCs) [23,25], and the hybrid sterility in interspecific crosses results in unfruitful or very low selfing rates [8]. The offspring of interspecific hybrids were often obtained through backcrossing [9], but this would inevitably reduce the variation of interspecific hybrids and fail to achieve the purpose of obtaining more genetic variation. Through SNP detection and genetic distance cluster analysis, the plants in the W58 line had rich genetic variation and were developing in a different evolutionary direction from T19-4. The three plants in the W59 line (W59-3, W59-4, W59-6) were more abundant in genetic variation than T19-5 and had different evolutionary direction from T19-5. The genetic distance between the induced offspring W59 and the parent was further than T19-5, the selfing offspring F_2_ (W97), backcross offspring BC_1_F_1_ (W99) and the parent (Figure 5). Induction of interspecific hybrid progeny through DH inducer could not only make the progeny part of the rapid stabile (Table S4), but also preserve or broaden the genetic variation of a single plant.

The results showed that DH induction lines could avoid the phenomenon of continuous backcrossing, making the genetic distance closer, enriching the genetic variation of the progeny, and making some of the progeny stabilize quickly. This plays an important role in expanding interspecific hybridization to obtain richer genetic variation.

### 3.4. The Important Role of DH Inducible Lines in Obtaining Interspecific Hybrid Progeny

DH inducer in *B. napus* was an octoploid rapeseed with special effects recently discovered by our research team [18,19]. The application of DH inducer in interspecific hybridization has not been reported yet. By studying the characteristics of octoploid induction system materials, it was found that octoploids were genetically unstable [27,31], prone to producing aneuploid gametes, and prone to mixed ploidy [22,27,31]. Combining the genesis mechanism of maize haploid induction line [32], it was found that the DH induction lines together determine the high inducibility due to genetic characteristics and ploidy [22]. At the same time, it was also found that a large number of aneuploid gametes appeared in triploid pollen. T19-4 and T19-5 could induce induction, which also provides a reference for other species to develop an induction phenomenon [22]. In this study, the selfing rate of triploid pollen was very low, and it did not induce its own egg cells. The main reason was that it was unable to induce itself due to hybrid sterility. Through the analysis of the SNP loci of the induced progeny and the induction mechanism of the DH line, it was speculated that the induction process was due to the zygotes produced by the sperm cells of the induction line and the maternal parent, with the paternal chromosome being eliminated during the mitosis of the zygote and maternal chromosomes or early embryonic chromosomes doubling to form DH offspring. The loss of the paternal (DH induction line) chromosome may be incomplete, there may be chromosome exchange or transposon jumping during the loss process, or homozygous SNP sites or fragments may come from the paternal parent. At the same time, the induction effect was not only related to the induced maternal nucleus, but also to its cytoplasmic genotype [21,22]. This was due to the genetic distance of the induced female parent which was closely related to the induction line. The chromosome loss of the genetic relationship close to the male parent was not prone to occur and was prone to hybridization or incomplete hybridization. The emergence of five hexaploid rapeseeds and one octaploid rapeseed in W58 and W59 illustrated this problem. However, DH inducer Y3380 and three hexaploid plants were not in the same cluster class as one octoploid plant, indicating that the male parent of Y3380 was less infiltrated and complicated genome changes might have occurred after hybridization, which requires further research. At the same time, according to the Y3380 breeding process, it had more sources of A chromosome [18]. Therefore, in this study, the highest rate of homozygous SNPs induced by F_1_ was only 81.63%, which may be related to the incomplete chromosome loss of the male parent of the induced line and the existence of partial hybridization. Although no DH with homozygous sites greater than 95% were obtained, this does not prove that the previous DH nomenclature does not apply, and it could not be denied that the offspring with stable traits were induced. 

In this study, the induction of maternal egg cells by the DH inducer was used to realize the self-breeding of egg cells (megaspores) of interspecies hybridization. The chromosomes of the inducible line were lost or incomplete lost after the interspecific hybridization F_1_ was crossed with the inducible line. Although the infiltration of chromosome fragments or partial genes of the induced line could not be completely excluded which prevented us from obtaining DH with a homozygosity rate greater than 95%, for interspecific hybridization more genetic variation was retained, which opened up a new way for the rapid acquisition of interspecific hybrid progeny.

## 4. Materials and Methods

### 4.1. Planting Material and Hybrid Combination

**Figure 6:**
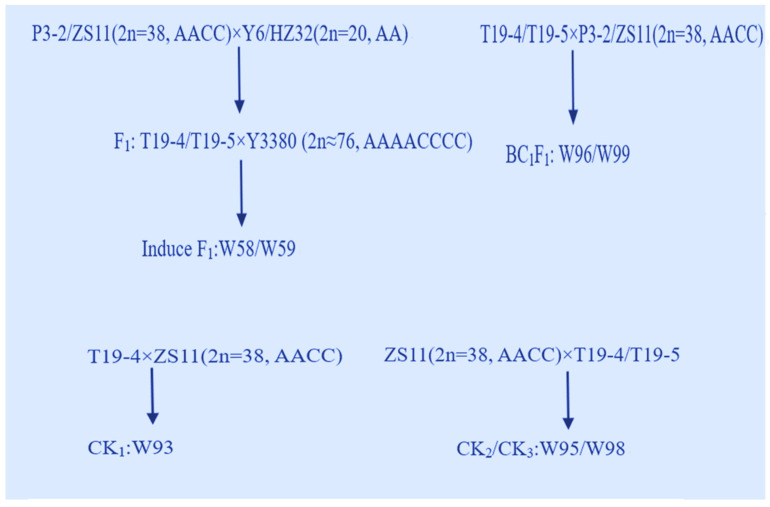
Schematic diagram of each hybrid combination.

Two samples of common *B. napus* were used in this study: P3-2 bred by Fu of Chengdu Academy of Agricultural and Forestry Sciences (*Brassica napus* L., AACC, 2n = 4x = 38) [18], the conventional variety ZS11 selected by the oil crop research of the Chinese Academy of Agricultural Sciences (*Brassica napus* L., AACC, 2n = 4x = 38). Two common *Brassica* campestris: Y6 (*Brassica rapa* L., AA, 2n = 2x = 20), HZ32 (*Brassica rapa* L., AA, 2n = 2x = 20) were also used. The octaploid Y3380 (2n = 8x ≈ 76, AAAACCCC) artificially synthesized by Fu of Chengdu Academy of Agricultural and Forestry Sciences in 2017 was a DH inducer in *B. napus* [18]. The above materials were provided by the Chengdu Branch of the National Rape Improvement Center of Chengdu Academy of Agricultural and Forestry Sciences. The above test materials of P3-2, ZS11, Y6, and HZ32 were uniformly spaced in October 2018 and sown evenly in the network room of Chengdu Academy of Agriculture and Forestry Sciences (E103.83, N30.70), and artificial pollination was carried out during the flowering period in March 2019 and F_1_ developed in May 2019. In October 2019, F_1_ was planted in the network room of Chengdu Academy of Agriculture and Forestry Sciences, with equal row spacing and even seeding. In March 2020, F_1_ was bagged and self-bred to obtain F_2_; F_1_ were pollinated with *Brassica* DH inducer Y3380 to induce F_1_; F_1_ was backcrossed with female parent P3-2 or ZS11 to obtain BC_1_F_1_, T19-4, and ZS11 hybridized obtained CK_1_, ZS11 and T19-4 hybridized obtained CK_2_, and ZS11 and T19-5 hybridized obtained CK_3_, ZS11 self-bred obtained CK_4_. The schematic diagram of each hybridization combination is shown in Figure 6. The names of all crossing and selfing combinations, as well as the progeny of the test materials, can be found in Table S1, Supplementary Materials.

### 4.2. Morphological Analysis

The morphological observation of this experiment mainly included the morphological characteristics of parents, F_1_ and induced F_1_, planted at the seedling stage, selecting representative plants after pod harvest, taking photos with SLR camera (Canon E0s 200D), and making statistics on the seed setting rate of F_1_, BC_1_F_1_, induced F_1_, and CK [21,22]. The pollen fertility of the parents, F_1_, and induced F_1_ was determined [33]. The pollen fertility statistics need to be gathered around 10 am on a sunny day. We took 3 newly opened flowers of the plant to be tested, and evenly spread the pollen on the glass slide with 1% acetic magenta stain with the dye solution, covered with a cover glass gently, then observed and counted under the × 20 lens of the microscope. We observed 5 fields of view and repeated 3 times. Pollen that was huge, round, and colored red was fertile pollen, whereas sterile pollen was small, deflated, and lighter in color. Pollen fertility % = fertile pollen number/total pollen number × 100. We adopted the Hoekstra [34] method to cultivate F_1_ and induced F_1_ pollen and calculated the germination rate after 4 h. As pollen germination occurred, the length of the pollen tube was larger than or equal to 2 times the diameter of the pollen grain. Germination rate percentage was calculated by following formula: germination rate % = germinated pollen number/total pollen number × 100.

### 4.3. Flow Cytometry to Identify Ploidy 

Flow cytometry has been widely used in the determination of DNA content [35,36]. In this experiment, flow cytometry was used to determine the DNA content of the parent, F_1_, and induced F_1_ to determine the ploidy according to the reference, from 8:00 to 11:00 am. We took the fresh young leaves of the tested plants, washed them with distilled water, and dried them with filter paper; avoided the main leaf veins, took the leaves with a hole punch with a diameter of 8 mm and placed them in a pre-cooled petri dish, and added 0.5 mL of pre-cooled cell lysis buffer (15 mM Tris, 2 mM disodium oxalate, 0.5 mM spermine tetrachloride, 80 mM potassium chloride, 20 mM sodium chloride, 0.1% Triton-100 and 15 mM β-mercapto ethanol, pH 7.5, filtered with 0.22 mM filter membrane). We used a sharp razor to quickly chop the leaves to very fine particles and filtered with a 300-mesh filter into a 2 mL EP tube. After that, 1.5 mL PI staining solution (5% propidium iodide and 5% RNase) was added in the dark for 30 min and then measured on the machine to collect 20,000 cell particles. Before testing the samples, we used ZS11 as a reference for calibration (ZS11 is a popularized variety, confirmed tetraploid *B. napus*), and used this as a reference for ploidy determination. We used a flow cytometer (Accuri C6 Plus, BD Biosciences, Franklin Lakes, NJ, USA) to measure the processed material. The PI-stained cell nucleus suspension entered the flow chamber and emitted fluorescence through laser irradiation. Then, BD AccuriC6 Plus was used to determine the fluorescence value (peak value) at the position of cell division G1. Sample ploidy = reference material ploidy × (fluorescence value (peak value) at the position of sample G1 peak/fluorescence value (peak value) at the position of G1 peak of reference material).

### 4.4. General Cytology Observation 

Mitosis was observed with the root tip. The rapeseed seeds were sterilized in 75% alcohol for 30 s at 9 a.m., 0.1% mercury liters for 5 min, and sterilized water was rinsed more than three times. The seeds were placed in a sterilized germination box and cultured in the dark at 25 °C for 24 h in an incubator. At 9 a.m. the next day, the germination box was placed in an incubator at 15 °C for 12 h in the dark, and at 9 p.m., the germination box was placed in an incubator at 25 °C for 12 h in the dark according to the method of Li [37] and Lang [38]. At 9 a.m. on the third day, we took a 1–2 cm root tip from the ultra-clean workbench and put the moist root tip into a 1.5 mL centrifuge tube (the cap was pierced with a red-hot dissecting needle) and put it in nitrous oxide for 2 h. Afterward, we added acetic acid to the centrifuge tube and fixed it for 5 min. We took out the root apex and washed it with deionized water, then cut the 1–5 mm root apical meristem, soaked up the water, and put 3 roots in 30 μL enzymatic hydrolysis solution (1% pectinase, pectolyase Y-23; 2% fiber enzymatic hydrolysis in cellulase Onozuka R-10) at 37 °C for 45 min, then rinse it with 70% alcohol for 3 times and added 100 μL 70% alcohol. We used a grinder to grind for 5–8 s. After grinding, we placed it in a centrifuge at 4500 r, centrifuged for 3 min, poured out the supernatant, added 60 μL acetic acid, vortexed and placed it in a humidified box for dropping the slides and dropped 10 μL on each slide. After air-drying, we added 10 μL DAPI dropwise and covered with a cover glass. After staining for 20 min, a microscopic examination could be performed. When observing meiosis, we took a flower bud with a diameter of about 2 mm at 10 am on a clear morning, treated it with 0.002 mol/L 8-antylquinoline at room temperature for 3–4 h, and fixed it with Carnoy’s solution (ethanol: glacial acetic acid = 3:1). After 24 h, we removed the anthers from the young buds with tweezers, rinsed them with deionized water 2–3 times, put them in a centrifuge tube containing 1 mol/L hydrochloric acid, and dissociated them in a 60 °C water bath for 2–3 min. After the dissociation was completed, the tablets were stained with 10% modified carbopol fuchsin and then subjected to meiosis observation.

### 4.5. Germination Dynamics of Pollen Tube in Silique 

We took the pistils of different cross combinations at 24 h and 48 h after pollination. We took 3 siliques from each material for three repetitions, fixed them with Carnoy’s solution (ethanol: acetic acid = 3:1) for more than 24 h, then transferred to 70% ethanol for 30 min, rinsed with distilled water for 30 min, then transferred to 1 M NaOH solution for softening treatment for 12 h, then We took the material out and rinsed it three times with distilled water, each time for ten minutes. We used 2% modified aniline blue dye solution (aniline blue (0.1 M K3PO4, pH 11), 1 M (pH 9.5) glycerin, and distilled water mixed in a volume ratio of 5:8:7) for 3–5 h [39] under complete darkness. We placed the stained pistil on a glass slide, removed the silique wall with tweezers and a dissecting needle, covered it with a cover glass and gently pressed the slide, then observed and took pictures under the ultraviolet light of a fluorescence microscope to observe the intensity of fluorescence on the *Brassica* stigma. We counted the fertilization rate 48 h after pollination. Fertilization rate percentage was calculated by the following formula: Fertilization rate % = the number of ovules with fluorescent dots in the silique/total number of ovules in the silique × 100.

### 4.6. Fluorescence In Situ Hybridization

The DNA special probe BNIH123L05 [40] in the BAC library containing the specific *Brassica* C genome was provided by Huazhong Agricultural University and had been introduced into the E. coli plasmid vector. The bacterial solution of E. coli was thawed and activated in LB medium (25 μg/mL chloramphenicol), and cultured at 37 °C. We shook the bacteria overnight and scribed to LB medium plate (25 μg/mL chloramphenicol), cultured overnight at 37 °C. A single colony of Escherichia coli was selected in liquid LB medium (25 μg/mL chloramphenicol) and cultured overnight at 37 °C. The DNA probe of the overnight culture broth was extracted using the Gold HiEndofree Plasmid Maxi Kit instruction method to extract the probe DNA of the specific rapeseed C genome sequence BNIH123L05 in the BAC library. The probe was labeled using the Nick Translation method [41] and the Tamra-CTP marks the C genome probe in red. Fluorescence in situ hybridization slide preparation was the same as the mitotic observation in ordinary cytological observation. After the slides were air-dried, they were placed in a UV crosslinker for cross-linking (125 mJ/cm^2^) twice, and then 10 μL hybridization solution [42] (labeled probe 1 μL + 4.5 μL 2SSC + 4.5 μL × TE) was dropped on each slide and covered with a cover glass. We put it in a metal humid box with moist paper towels, heated and denatured in boiling water for 5 min, then hybridized in a thermostat at 42 °C for 14–16 h. After the hybridization was completed, we put the slide glass in a 2 × SSC preheated overnight at 42 °C to wash off the cover glass, then rinsed the slide glass with distilled water, dried the slide glass, dropped 7.5 μL DAPI, and placed it on the fluorescence microscope after 20 min Image acquisition was carried out under.

### 4.7. Analysis of Homozygous Rate and Genetic Distance of SNP Loci

SNP chip detection was completed by Wuhan Shuang lv yuan Research Institute, using *Brassica* 50K SNP (single nucleotide polymorphism) chip (developed jointly by Wuhan Shuang lv yuan Research Institute and Huazhong Agricultural University, professionally customized by Illumina Infinium) to extract parents, F_1_, induced F_1_, BC_1_F_1_, *Brassica* DH inducer Y3380. The genomic DNA was sent to Wuhan Shuang lv yuan Research Institute for final data analysis. According to the theta value of the sample, the genotypes were classified into three types: AA, BB, and AB. AA and BB represent two homozygous genotypes, and AB represents a heterozygous genotype. Based on the *B. napus* genome Darmor4, the frame of each chromosome was constructed. The ordinate was the length of the chromosome (Mb), and the abscissa was the chromosome name (the corresponding random chromosome on the right). We marked the sites with heterozygous genotypes (AB genotype) in blue on the map (the degree of homozygosity for each sample = the number of homozygous sites/total number of sites × 100%). By comparing the loci difference between the two samples, an *n* × *n* matrix was obtained, and the Test Maximum L of MEGA 11 software was used to calculate the Nei genetic distance and construct the genetic cluster map.

## 5. Conclusions

In this study, pollen fertility statistics, pollen vitality statistics, and pollen mother cell meiosis observations were performed on two pairs of hybridization between *B. napus* and *B. napus*. It was found that the disorder of pollen mother cell meiosis easily produces abnormal gametes, which leads to the low seed setting rate of selfing offspring. After backcrossing, self-crossing, and induction, the average fertilization rate and average seed setting rate of the offspring of hybridization between *B. napus* and *B. napus* were calculated. The genetic stability of egg cells was greater than that of sperm cells, which laid the foundation for DH induction. In view of this result, the DH inducer was used to induce the chromosomes of the egg cell (AC) to double which formed the self-progeny of the egg cell. Induced offspring could form tetraploid *B. napus* (2n = 4x = 38, AACC). At the same time, the genetic distance between some individual plants and the parent was greater than that of hybrid F_1_, backcross offspring, and the genetic distance between selfing offspring and parent, which enriched the genetic variation of offspring. This research provided a new insight and perspective for interspecific hybridization of rapeseed.

## Figures and Tables

**Table 1:** Table of average fertilization rate and average seed setting rate of each cross combination in F_1_.

Cross Combination	Average Fertilization Rate (the Number of Ovules with Fluorescent Dots in the Silique/Total Number of Ovules in the Silique) %	Average Effective Seed Setting Rate (the Number of Seeds/ the Number of Effective Siliques × 25) %
ZS11 × T19-4	(10/43) 23.26	(330/(115 × 25)) 11.48
ZS11 × T19-5	(9/34) 26.47	(40/(21 × 25)) 7.62
T19-4 × ZS11	(35/58) 60.34	(216/(38 × 25)) 22.74
T19-5 × ZS11	(45/65) 69.23	(173/(29 × 25)) 23.86
T19-4 × Y3380	(47/56) 83.93	(86/(13 × 25)) 26.46
T19-5 × Y3380	(28/38) 73.68	(92/(18 × 25)) 20.44
T19-4 ⊗	(3/48) 6.25	(13/(13 × 25)) 4.00
T19-5 ⊗	(5/42) 11.90	(35/(27 × 25)) 5.19
ZS11 ⊗	(48/53) 90.57	(402/(32 × 25)) 50.25
W58 ⊗	No data	(572/(78 × 25)) 29.33
W59 ⊗	No data	(313/(32 × 25)) 39.13

Note: about 25 ovules per pod, ⊗ was selfing.

**Table 2:** Table of ploidy numbers and ranges of flow cytometry values for each material.

Sample Number	Total Number of Plants	Peak Range (D Thousand Lines)	Average Peak (D Thousand Lines)	Number of Tetraploid Plants	Number of Triploid Plants	Number of Other Ploidy Plants
P3-2	3	482.62–498.71	493.36	3	0	0
Y6-1	3	261.16–313.41	287.18	0	0	2
ZS11	3	495.89–510.69	502.73	3	0	0
HZ32	3	243.76–303.69	274.48	0	0	2
T19-4	27	312.12–482.46	351.02	1	26	0
T19-5	23	307.29–493.68	349.64	1	22	0
W58	18	421.45–661.17	531.65	15	0	3
W59	6	478.27–1037.64	676.33	3	0	3

## Data Availability

The data presented in this study are available in article or Supplementary Materials.

## Supplementary Materials

The following supporting information can be downloaded at: https://www.mdpi.com/article/10.3390/plants11050695/s1, Figure S1: Observation diagram of induced F_1_ plant phenotype: (a) W58-1, (b) W58-2; (c) W58-3; (d) W58-6; (e) W58-7; (f) W58-8; (g) W58-12; (h) W58-13; (i)W58-14; (j) W58-15; (k) W59-1; (l) W59-2; (m) W59-3; (*n*) W59-4. These photos were taken in the same period. It could be seen that the morphological characteristics of induced F_1_ were different, and it could also indicate that the genetic distance was widened. The leaves of W58-8 resembled B. rapa, and early maturing, but it was a tetraploid *Brassica* napus; Figure S2: Homozygous site map of some samples, and the heterozygous sites was marked in blue: (a) W59-3; (b) W59-6; (c) W58-14; (d) W59-3; Table S1: Information table of all combinations of test materials and names of descendants; Table S2: The fertility and pollen tube germination rate statistics of parents, interspecific cross F_1_ and induced F_1_; Table S3: Flow cytometry identification results; Table S4: Induced F_1_ SNP site statistics.
