# Supplementary figures and images for: Rapid Creation of Interspecific Hybrid Progeny to Broaden Genetic Distance through Double Haploid (DH) Inducer in Brassica napus
Source: Plants (Basel). 2022 Mar 4;11(5):695. doi: 10.3390/plants11050695 (PMC8912716; doi:10.3390/plants11050695)

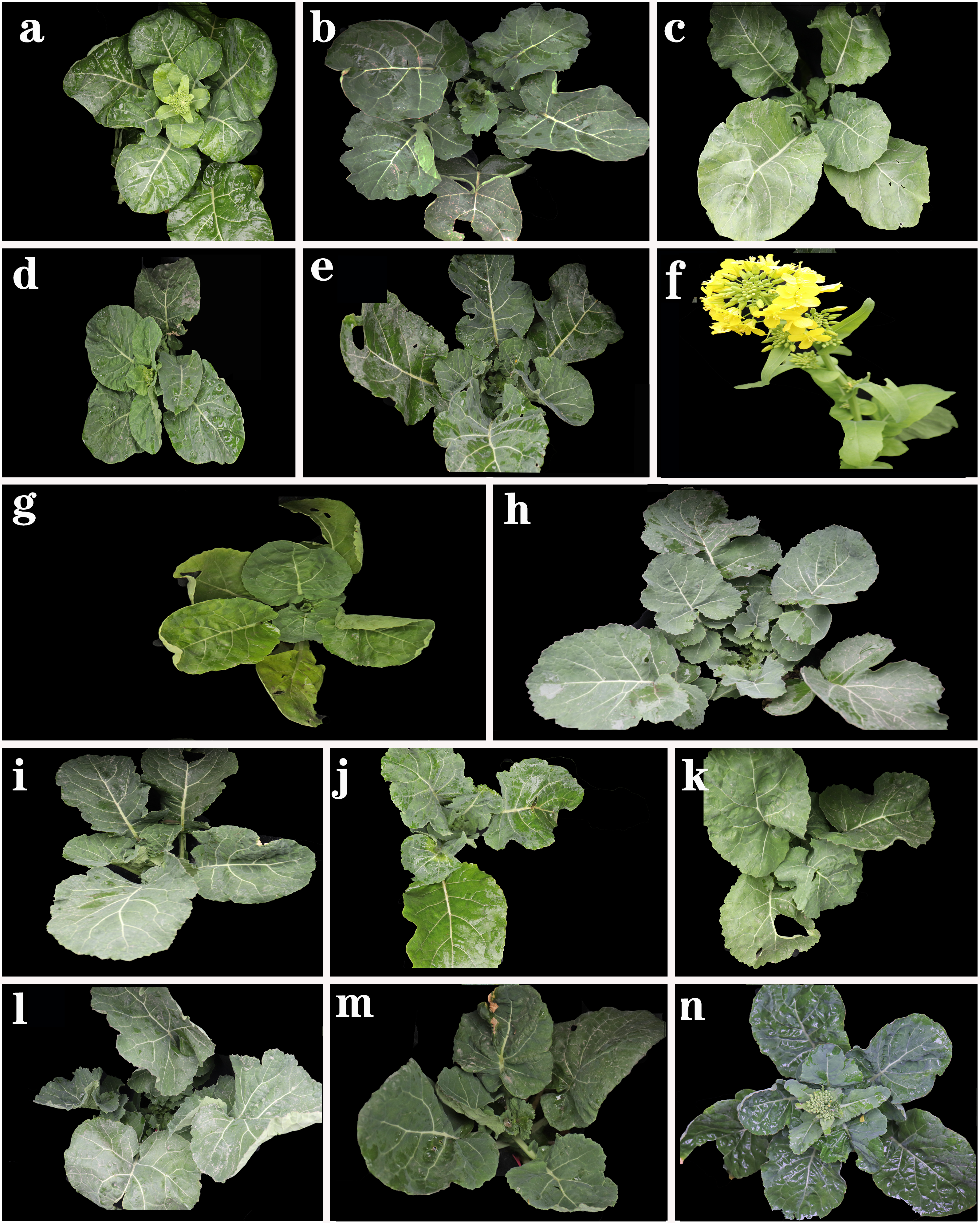

Supplement: Supplementary file 1 [file plants-11-00695-s001.zip › Figure S1 Observation diagram of induced F1 plant phenotype.tif]
